# Treatment Outcomes Among Pregnant Patients With Multidrug-Resistant Tuberculosis: A Systematic Review and Meta-analysis

**DOI:** 10.1001/jamanetworkopen.2022.16527

**Published:** 2022-06-10

**Authors:** Kefyalew Addis Alene, Megan B. Murray, Brittney J. van de Water, Mercedes C. Becerra, Kendalem Asmare Atalell, Mark P. Nicol, Archie C. A. Clements

**Affiliations:** 1 Telethon Kids Institute, Nedlands, Western Australia, Australia; 2 Faculty of Health Sciences, Curtin University, Bentley, Western Australia, Australia; 3 Department of Global Health and Social Medicine, Harvard Medical School, Boston, Massachusetts; 4 Connell School of Nursing, Boston College, Chestnut Hill, Massachusetts; 5 College of Medicine and Health Sciences, University of Gondar, Gondar, Ethiopia; 6 Institute for Infectious Diseases and Molecular Medicine, Division of Medical Microbiology, University of Cape Town, Cape Town, South Africa; 7 School of Biomedical Sciences, University of Western Australia, Perth, Western Australia, Australia

## Abstract

**Importance:**

The management of multidrug-resistant tuberculosis (MDR-TB) during pregnancy is challenging, yet no systematic synthesis of evidence has accurately measured treatment outcomes.

**Objective:**

To systematically synthesize treatment outcomes and adverse events among pregnant patients with MDR-TB.

**Data Sources:**

PubMed, Scopus, Web of Science, and ProQuest were searched from the inception of each database through August 31, 2021.

**Study Selection:**

Studies containing cohorts of pregnant patients with a defined treatment outcome were eligible.

**Data Extraction and Synthesis:**

Independent reviewers screened studies and assessed the risk of bias. The study followed the Preferring Reporting Items for Systematic Review and Meta-analyses reporting guideline. Meta-analysis was performed using random-effects models. The sources of heterogeneity were explored through metaregression.

**Main Outcomes and Measures:**

The primary outcome was the proportion of patients with each treatment outcome (including treatment success, death, loss to follow-up, and treatment failure), and the secondary outcomes included the proportion of patients experiencing adverse events during pregnancy.

**Results:**

In this systematic review and meta-analysis, 10 studies containing 275 pregnant patients with available data on treatment outcomes were included. The pooled estimate was 72.5% (95% CI, 63.3%-81.0%) for treatment success, 6.8% (95% CI, 2.6%-12.4%) for death, 18.4% (95% CI, 13.1%-24.2%) for loss to follow-up, and 0.6% (95% CI, 0.0%-2.9%) for treatment failure. Treatment success was significantly higher in studies in which the proportion of patients taking linezolid was greater than the median (20.1%) compared with studies in which this proportion was lower than the median (odds ratio, 1.22; 95% CI, 1.05-1.42). More than half of the pregnant patients (54.7%; 95% CI, 43.5%-65.4%) experienced at least 1 type of adverse event, most commonly liver function impairment (30.4%; 95% CI, 17.7%-45.7%), kidney function impairment (14.9%; 95% CI, 6.2%-28.3%), hypokalemia (11.9%; 95% CI, 3.9%-25.6%), hearing loss (11.8%; 95% CI, 5.5%-21.3%), gastrointestinal disorders (11.8%; 95% CI, 5.2%-21.8%), psychiatric disorders (9.1%; 95% CI, 2.5%-21.6%), or anemia (8.9%; 95% CI, 3.6%-17.4%). The pooled proportion of favorable pregnancy outcomes was 73.2% (95% CI, 49.4%-92.1%). The most common types of adverse pregnancy outcomes were preterm birth (9.5%; 95% CI, 0.0%-29.0%), pregnancy loss (6.0%; 95% CI, 1.3%-12.9%), low birth weight (3.9%; 95% CI, 0.0%-18.7%), and stillbirth (1.9%; 95% CI, 0.1%-5.1%). Most of the studies had low-quality (3 studies) or medium-quality (4 studies) scores.

**Conclusions and Relevance:**

In this systematic review and meta-analysis, high treatment success and favorable pregnancy outcomes were reported among pregnant patients with MDR-TB. Further research is needed to design shorter, more effective, and safer treatment regimens for pregnant patients with MDR-TB.

## Key Points

**Question:**

What are the treatment outcomes and adverse events among pregnant patients with multidrug-resistant tuberculosis (MDR-TB)?

**Findings:**

In a systematic review and meta-analysis of 10 studies including 275 pregnant patients with MDR-TB, the pooled proportion of treatment success was 72.5%, and the pooled proportion of favorable pregnancy outcomes was 73.2%. Adverse events, such as liver function impairment, kidney function impairment, hearing loss, and hypokalemia, were common among pregnant patients with MDR-TB, occurring in more than half of the patients.

**Meaning:**

This study suggests that a high rate of treatment success and favorable pregnancy outcomes can be achieved when pregnant patients with MDR-TB are treated with effective regimens.

## Teaser

This systematic review and meta-analysis examines treatment outcomes and adverse events among pregnant patients with multidrug-resistant tuberculosis (MDR-TB).

## Introduction

Multidrug-resistant tuberculosis (MDR-TB) is a major public health concern affecting approximately half a million people globally, but only 57% of cases of MDR-TB were successfully treated in 2019.^1^ The global burden of MDR-TB among pregnant patients is not well known, but pregnant patients are recognized as a population that is particularly vulnerable to MDR-TB.^2^ The management of MDR-TB among pregnant patients is particularly challenging and complex owing to the teratogenic effects of some of the second-line TB medications. The physiological dynamics of pregnancy might affect pharmacokinetic parameters that could lead to inadequate treatment and poor outcomes.^3^ A recent systematic review showed that MDR-TB is associated with a high risk of adverse maternal and perinatal outcomes, such as maternal death, pregnancy loss, preterm birth, and low birth weight.^4^

Although untreated MDR-TB is associated with significant maternal mortality, morbidity, and adverse pregnancy outcomes, there is no standardized regimen for treatment of MDR-TB among pregnant patients. There are currently limited options for second-line TB drugs for the treatment of pregnant patients with MDR-TB. World Health Organization (WHO) guidelines on the management of drug-resistant TB during pregnancy recommend the use of individualized, longer regimens containing at least 4 effective drugs with an established safety profile and low teratogenic risk.^5^ However, the adverse effects of these drugs on pregnancy outcomes have yet to be comprehensively quantified. Moreover, the treatment outcomes among pregnant patients with MDR-TB have not been well studied, with only a few observational studies available. The findings from these studies have provided inconclusive evidence. Therefore, we conducted a systematic review and meta-analysis of the available evidence on treatment outcomes among pregnant patients with MDR-TB. Our primary objective was to quantify the pooled proportion of patients with each treatment outcome, including treatment success, death, loss to follow-up, and treatment failure. Our secondary objective was to assess adverse pregnancy outcomes and adverse drug events. In addition, we aimed to assess study characteristics and TB medicines that may be associated with outcomes, particularly treatment success.

## Methods

This systematic review and meta-analysis followed the Preferred Reporting Items for Systematic Reviews and Meta-analyses (PRISMA) reporting guideline.^6^ The review protocol has been registered in PROSPERO (CRD42021273215).^7^

### Search Strategy

We searched for publications in the PubMed, Scopus, Web of Science, and ProQuest databases from the inception of each database through August 31, 2021, without restrictions on language or year of publication. We used relevant MeSH headings and keywords for MDR-TB, treatment outcomes, and pregnancy (eTable 1 in the Supplement). Reference lists of all included studies were also assessed to ensure that relevant studies were not missed. When articles could not be accessed, we contacted authors by email to request the full texts.

### Study Selection Criteria

Studies were eligible for inclusion if they reported MDR-TB treatment outcomes among pregnant patients. Studies conducted only on drug-susceptible TB or animal studies were excluded. We also excluded abstracts, case reports, correspondence, reviews, editorials, and duplicate studies. Case series with fewer than 5 pregnant patients were also excluded.

### Outcomes of the Study

The primary outcome of interest was treatment outcome, which includes treatment success, treatment failure, death, and loss to follow-up. Treatment success was defined as a sum of cure and treatment completion (eTable 2 in the Supplement).^8^ Similar definitions were used by previous systematic reviews.^9,10^ Secondary outcomes of the study were drug-related adverse events and pregnancy outcomes, such as preterm birth, miscarriage, neonatal death, stillbirth, and low birth weight.

### Screening of Articles

Duplicates were first removed from all citations identified through our search strategy. Two of us (K.A. Alene and K.A. Atalell) independently screened the titles, abstracts, and full texts to identify eligible studies. Any discrepancies were discussed and resolved by consensus. EndNote, version X8 (Clarivate) and Rayyan (Rayyan)^11^ were used to assist with screening.

### Data Extraction and Quality Assessment

Data were extracted from included articles using a piloted form. We collected information about the characteristics of patient cohorts, studies, and outcomes of interest. The quality of the included studies and the risk of bias were assessed by the same 2 researchers (K.A. Alene and K.A. Atalell) using the Newcastle-Ottawa Scale.^12^ The tool has scores ranging from 0 to 9 with low-quality (0-4), medium-quality (5-7), and high-quality (8-9) groupings (eTable 3 in the Supplement).

### Statistical Analysis

Meta-analysis was performed using a random-effects model to estimate the pooled proportion of treatment outcomes, pregnancy outcomes, or adverse events. To account for the sample size differences between the studies, we conducted the meta-analysis using inverse variance weights. The inverse variance weight represented a composite measure of total variances and sample size such that studies with larger sample sizes were given more weight than smaller studies. This choice of weights minimizes the uncertainty of the pooled effect estimate. Analyses were performed separately for each of the outcomes when 2 or more studies were available on the outcome of interest. The summary effect estimates and 95% CIs for individual outcomes were represented with a forest plot. Heterogeneity between studies was examined using the Cochran *Q* test and quantitatively measured by the index of heterogeneity squared (*I*^2^) statistics and corresponding 95% CIs.^13^ Heterogeneity was considered low when *I*^2^ values were below 25%, moderate when *I*^2^ values were between 25% and 75%, or high when *I*^2^ values were above 75%.^13^ The sources of heterogeneity were explored through metaregression using study characteristics as covariates. The WHO grouping of medicines recommended for use in longer MDR-TB regimens was also used as a covariate (eTable 4 in the Supplement).^8^ Potential publication bias was assessed using funnel plots, and asymmetry was evaluated using the Egger method. The significance level was set at *P* < .05, and all *P* values were 2-tailed. All statistical analyses were performed using Stata software, version 16 (StataCorp LLC).

## Results

### Study Selection

**Figure 1.**
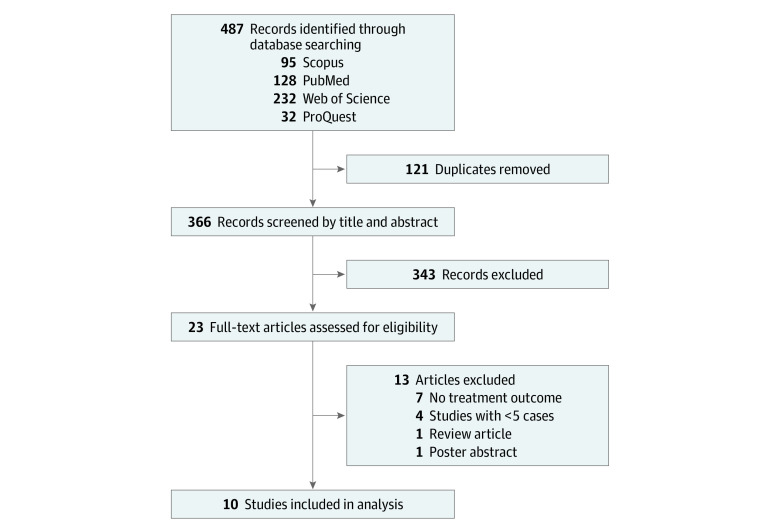
Study Selection Process for the Systematic Review and Meta-analysis

Our electronic database searches identified 487 records. After removal of duplicates, excluding studies by title and abstract screening and full-text review (eTable 5 in the Supplement), 10 studies^14,15,16,17,18,19,20,21,22,23^ were included in the meta-analysis, which included 288 pregnant patients with MDR-TB (Figure 1).

### Study Characteristics

The studies were conducted in 5 different countries, and the data were collected from 1996 to 2019 (eTable 6 in the Supplement). Among the included studies, 9 were published in English,^14,15,16,17,18,19,20,21,22^ and 1 was in Spanish.^23^ Treatment outcomes were reported for all 10 studies, which included 275 of the 288 pregnant patients with MDR-TB (Table 1). End-of-treatment outcomes were unknown for 13 patients who had transferred out of care or were receiving treatment at the time of publication; these patients were excluded from the meta-analysis. Data were available on successful treatment outcomes for 192 patients from all studies. There were 22 deaths, 7 treatment failures, and 49 losses to follow-up, all reported from 9 studies (1 study^19^ reported overall poor treatment outcome for 5 patients without specifying whether the outcome was death, treatment failure, or loss to follow-up).

**Table 1.** Treatment Outcomes of Pregnant Patients With Multidrug-Resistant Tuberculosis

Source	Sample size, No.	Sample, No.^a^	Cure, No.	Completed treatment, No.	Death, No.	Treatment failure, No.	Loss to follow-up, No.	Successful outcome, No.^b^	Poor outcome, No.^c^	Unknown treatment outcome, No.^d^
Mokhele et al,^14^ 2021	35	28	4	13	3	0	8	17	11	7
Loveday et al,^15^ 2021	108	108	58	14	8	3	25	72	36	NA
Baluku and Bongomin,^16^ 2021	18	18	14	1	1	0	2	15	3	NA
van der Walt et al,^17^ 2020	26	26	17	0	1	1	7	17	9	NA
van de Water et al,^18^ 2020	8	8	5	2	0	0	1	7	1	NA
Azeez et al,^19^ 2018	36	36	NA	NA	NA	NA	NA	31	5	NA
Tabarsi et al,^20^ 2011	5	5	5	0	0	0	0	5	0	NA
de Oliveira and Mateus,^23^ 2011	7	6	2	0	3	0	1	2	4	1
Palacios et al,^21^ 2009	38	35	23	0	5	2	5	23	12	3
Shin et al,^22^ 2003	7	5	3	0	1	1	0	3	2	2
Total	288	275	131	30	22	7	49	192	83	13

Abbreviation: NA, not applicable.

^a^ Number of pregnant patients whose treatment outcomes were reported.

^b^ Successful outcome was the sum of cure and treatment completed.

^c^ Poor outcome was the sum of death, failure, and loss to follow-up.

^d^ Unknown treatment outcomes due to transfer out (9 patients) or current receipt of treatment (4 patients); these patients were excluded from the pooled analysis.

### Treatment Outcomes

The overall pooled proportion of patients achieving treatment success was 72.5% (95% CI, 63.3%-81.0%; *I*^2^ = 44.7%; *P* = .06) (eFigure 1 in the Supplement). The pooled proportion of pregnant patients who died was 6.8% (95% CI, 2.6%-12.4%; *I*^2^ = 20.5%; *P* = .26) (eFigure 2 in the Supplement). eFigure 3 in the Supplement shows the pooled proportion of treatment failure, which was 0.6% (95% CI, 0.0%-2.9%; *I*^2^ = 0.0; *P* = .77). The pooled proportion of pregnant patients whose outcome was loss to follow-up was 18.4% (95% CI, 13.1%-24.2%; *I*^2^ = 0.0; *P* = .54) (eFigure 4 in the Supplement).

### Treatment Outcomes by Study Characteristics

The pooled proportion of treatment success did not differ significantly by study characteristics (Table 2). However, loss to follow-up was significantly lower in studies conducted in Peru than in South Africa (odds ratio [OR], 0.88; 95% CI, 0.82-0.94) and in studies conducted before 2010 than in studies conducted after 2010 (OR, 0.89; 95% CI, 0.82-0.96) (eTable 7 in the Supplement). Furthermore, loss to follow-up was higher in studies with high HIV prevalence (ie, above the median value of 50%) than in studies with low HIV prevalence (OR, 1.11; 95% CI, 1.06-1.17) and was higher in studies with pregnant patients aged 26 to 30 years than in studies with pregnant patients aged 21 to 25 years (OR, 1.11; 95% CI, 1.03-1.21). The pooled proportion of patients who died during treatment was also higher in studies that included patients with both pulmonary TB (PTB) and extrapulmonary TB (EPTB) than in studies that included only patients with PTB (OR, 1.07; 95% CI, 1.01-1.13) (eTable 7 in the Supplement).

**Table 2.** Pooled Proportion of Treatment Outcomes of Pregnant Patients With Multidrug-Resistant TB by Study Characteristics

Category	Treatment success		Studies, No.	Pooled proportion (95% CI), %		
	Studies, No.	Pooled proportion (95% CI), %		Death	Treatment failure	Lost to follow-up
Country						
South Africa	4	70.4 (59.0-80.7)	3	6.9 (3.2-11.7)	1.8 (0.1-5.0)	24.4 (17.9-31.5)
Peru	3	70.0 (55.0-83.4)	3	9.6 (1.5-21.3)	3.4 (0.0-12.8)	10.1 (1.9-22.1)
Others^a^	3	77.9 (38.2-100)	3	12.0 (0.0-45.0)	0.0 (0.0-5.7)	8.1 (0.0-23.5)
Year of data collection						
2010-2019	6	73.6 (63.9-82.3)	5	5.7 (2.3-10.0)	0.9 (0.0-3.5)	22.0 (16.0-28.6)
1996-2009	4	67.8 (40.9-90.3)	4	15.9 (2.1-35.9)	2.7 (0.0-11.6)	8.4 (1.0-19.8)
Median age, y^b^						
21-25	4	67.8 (40.9-90.3)	4	15.9 (2.1-35.9)	2.7 (0.0-11.6)	8.4 (1.0-19.8)
26-30	6	73.6 (63.9-82.3)	5	5.7 (2.3-10.0)	0.9 (0.0-3.5)	22.0 (16.0-28.6)
Body sites						
PTB only	3	68.3 (60.0-76.1)	3	4.8 (1.3-9.6)	1.4 (0.0-5.0)	22.3 (15.3-29.9)
Both PTB and EPTB	2	63.5 (51.0-75.2)	2	12.6 (5.2-22.4)	2.2 (0.0-8.1)	20.2 (10.9-31.3)
Unknown	5	79.4 (58.9-95.0)	4	13.0 (0.0-38.0)	0.1 (0.0-8.1)	6.1 (0.0-19.5)
Previous TB treatment						
≤50%	3	67.3 (59.1-75.2)	3	6.0 (2.1-11.2)	0.6 (0.0-3.6)	22.7 (15.7-30.3)
>50%	3	70.0 (59.0-80.0)	3	8.3 (2.7-15.9)	3.2 (0.1-9.0)	17.2 (9.3-26.8)
Unknown	4	76.4 (44.9-98.2)	3	19.7 (0.0-56.6)	3.0 (0.0-21.9)	3.4 (0.0-22.8)
HIV			3			
≤50%	3	66.9 (43.7-86.9)	3	15.5 (1.7-36.4)	1.5 (0.0-8.1)	12.4 (4.2-23.2)
>50%	4	70.4 (59.0-80.7)	3	6.9 (3.2-11.7)	1.8 (0.1-5.0)	24.4 (17.9-31.5)
Unknown	3	86.8 (60.2-100)		2.2 (0.0-18.9)	2.2 (0.0-18.9)	3.2 (0.0-20.9)

Abbreviations: EPTB, extrapulmonary tuberculosis; PTB, pulmonary tuberculosis; TB, tuberculosis.

^a^ Includes Brazil, Iran, and Uganda.

^b^ The median age of pregnant patients was not available for 1 study; therefore, we took the median age of the whole sample (37 years).

**Figure 2.**
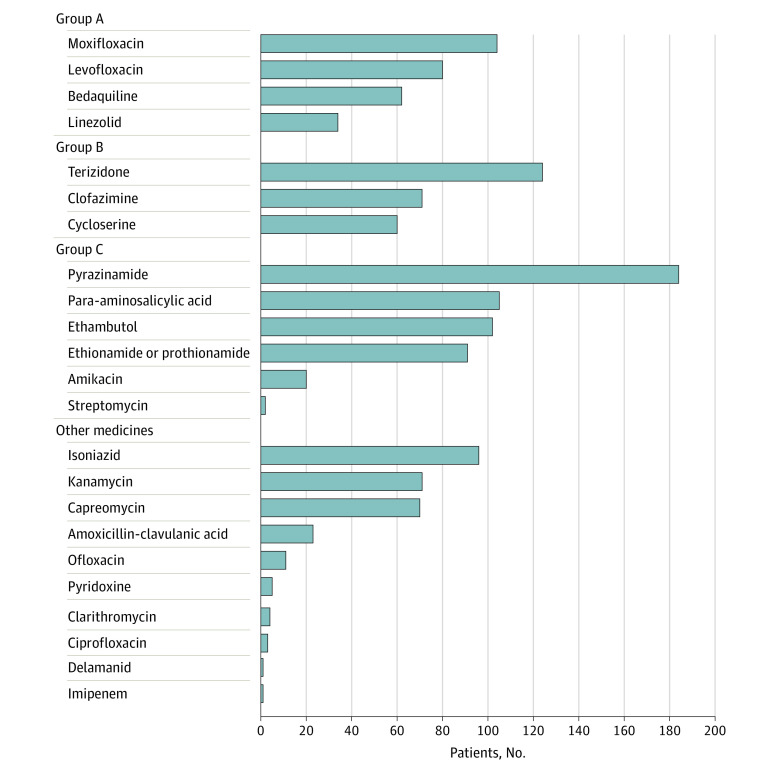
Tuberculosis Medicines Used by Pregnant Patients Based on the World Health Organization Grouping^8^

Eight studies^14,15,16,17,18,20,21,22^ documented the different drug regimens used for the treatment of MDR-TB (Figure 2). The most common drugs used included pyrazinamide, cycloserine, prothionamide, and fluoroquinolones. Linezolid-containing regimens were reported in 4 studies,^14,15,16,18^ and the percentage of pregnant patients taking linezolid in these studies ranged from 3.2% to 62.5% (median, 12.1%). Treatment success was significantly higher in studies with a higher percentage (ie, above the median value of 20.1%) of linezolid-containing regimens (85.0%; 95% CI, 67.4%-97.3%) than in studies with a lower percentage of linezolid-containing regimens (65.6%; 95% CI, 57.3%-73.5%; *P* = .03; OR, 1.22; 95% CI, 1.05-1.42). Treatment success rates did not differ by other TB medicines (eTable 8 in the Supplement).

### Adverse Events

Drug-related adverse events were reported in 4 studies (86 patients)^15,16,17,22^; 45.3% (95% CI, 34.5%-56.4%) of patients had no reported adverse events, whereas 54.7% (95% CI, 43.5%-65.4%) of patients experienced at least 1 type of adverse event (eTable 9 in the Supplement). The most common adverse events reported were liver function impairment (30.4%; 95% CI, 17.7%-45.7%), kidney function impairment (14.9%; 95% CI, 6.2%-28.3%), hypokalemia (11.9%; 95% CI, 3.9%-25.6%), hearing loss (11.8%; 95% CI, 5.5%-21.3%), and gastrointestinal disorders (11.8%; 95% CI, 5.2%-21.8%). Other serious adverse events were psychiatric disorders (9.1%; 95% CI, 2.5%-21.6%) and anemia (8.9%; 95% CI, 3.6%-17.4%).

### Pregnancy Outcomes

**Figure 3.**
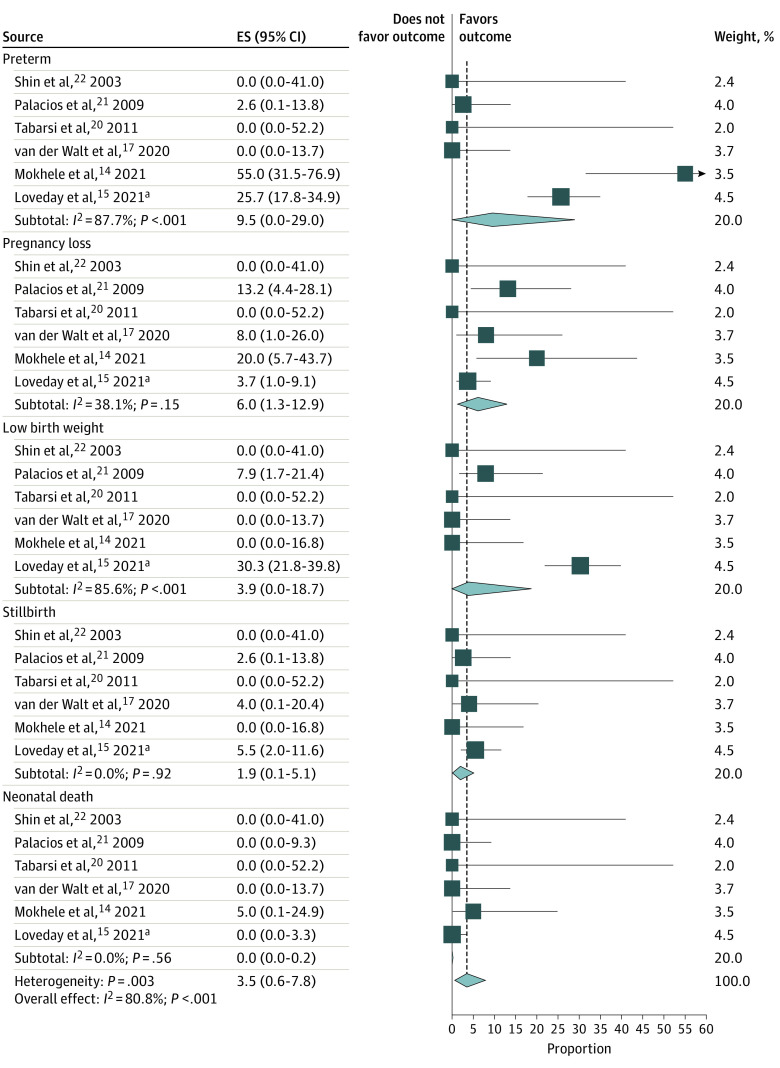
Pooled Proportion of Pregnancy Outcomes Among Patients With Multidrug-Resistant Tuberculosis The dashed vertical line indicates the overall pooled effect estimate (ES). Diamonds indicate pooled mean prevalence estimates; the extremes of the diamonds indicate 95% CIs. Horizontal whiskers indicate lowest to highest point prevalence estimates. The size of the squares refers to the proportional weight of each study. ^a^This study had 108 patients with 109 fetuses, including 1 set of twins.

Six studies reported on pregnancy outcomes^14,15,17,20,21,22^ (eTable 10 in the Supplement). The pooled proportion of favorable pregnancy outcomes was 73.2% (95% CI, 49.4%-92.1%). The most common types of adverse pregnancy outcomes were preterm birth (9.5%; 95% CI, 0.0%-29.0%), pregnancy loss (6.0%; 95% CI, 1.3%-12.9%), low birth weight (3.9%; 95% CI, 0.0%-18.7%), and stillbirth (1.9%; 95% CI, 0.1%-5.1%) (Figure 3).

### Quality Assessment

The overall score for the studies was between 3 and 8 out of a possible score of 9 (eTable 11 in the Supplement). Three studies had low quality, 4 studies had medium quality, and 3 studies had relatively high quality. There was no publication bias for successful treatment outcome (bias coefficient, 0.16; 95% CI, –0.89 to 1.23; *P* = .72) (eFigure 5 in the Supplement).

## Discussion

To our knowledge, this is the first comprehensive systematic review and meta-analysis of treatment outcomes among pregnant patients with MDR-TB. Our systematic review identified only 10 eligible studies reporting treatment outcomes of MDR-TB, including 275 pregnant patients with available data. Studies of MDR-TB among pregnant patients remain inadequate, and more data are needed to inform treatment options for pregnant patients with MDR-TB.

### Treatment Outcomes

The overall pooled treatment success among pregnant patients with MDR-TB was 72.5%, which is close to the WHO target of 75% and lower than the findings from previous systematic reviews and meta-analyses on treatment success among children with MDR-TB (77.0%-83.4%).^9,24,25,26,27^ Although the studies did not include comparison data for nonpregnant patients or patients with drug-susceptible TB, the treatment success rate in our study was higher than the treatment success rate reported by previous systematic reviews among adults (60%-69%).^10,28,29,30,31,32,33^ Given the limited drug options available to treat individuals with MDR-TB, especially pregnant patients, the high proportion of patients with treatment success in our study compared with previously reported findings in the general adult population is encouraging.

The use of recently recommended novel drugs—including bedaquiline, delamanid, and linezolid—for the treatment of MDR-TB during pregnancy may increase the treatment success rate and help to achieve the WHO targets.^8^ Our subgroup analysis found that the treatment success rate was significantly higher for studies with a higher percentage of linezolid-containing regimens (85.0%) than for studies with a lower percentage of linezolid-containing regimens (65.6%). However, this finding might be confounded by the use of other drugs, such as bedaquiline and delamanid, because the associations of these drugs with treatment outcomes were not assessed because of the small number of studies in which they were used. Previous systematic reviews and meta-analyses showed that linezolid was significantly associated with higher treatment success rates and lower mortality rates among adult patients with MDR-TB.^29,34^ All of these findings indicate that linezolid might be used to effectively treat pregnant patients with MDR-TB. However, a meta-analysis of individual participant data would be required to assess the independent association of each drug, including linezolid, with treatment success. Linezolid cannot be used for long periods because of hematologic adverse effects. Further research on the safety, tolerability, timing, and duration of linezolid, bedaquiline, and delamanid treatment during pregnancy, using adequately powered studies, is a priority.

Although the pooled proportion of patients experiencing treatment failure was low (0.6%), the pooled proportion of loss to follow-up was high (18.4%). This finding suggests that loss to follow-up was a major challenge to achieving a high rate of treatment success among pregnant patients with MDR-TB, which is consistent with the rates of loss to follow-up among other populations with MDR-TB (12%-17%).^10,28,29,30,31,32,33^ The loss to follow-up problem among pregnant patients could be mitigated with more integrated care because pregnant patients often access medical care during pregnancy. Our metaregression showed that the pooled proportion of loss to follow-up was significantly associated with high HIV prevalence and older age. This finding is consistent with previous studies reporting that HIV coinfection was associated with a high rate of loss to follow-up among patients with MDR-TB.^27,35^ This finding could occur because loss to follow-up includes unrecorded deaths. Adopting and implementing a shorter MDR-TB treatment regimen (ie, <12 months) recently recommended by the WHO might be a solution to reduce the high rate of loss to follow-up.^8^ A previous systematic review conducted to identify strategies for reducing loss to follow-up showed that engagement of community health workers as treatment professionals and the provision of patient education was associated with lower rates of loss to follow-up.^36^ Financial support and psychological counseling were also reported as important interventions associated with reducing the rate of loss to follow-up and treatment failure.^8^

Our systematic review and meta-analysis also showed that the pooled proportion of patients with MDR-TB who died during treatment was 6.8%. This finding is consistent with results from previous systematic reviews on adults and children with MDR-TB.^9,10,33^ More important, our metaregression indicated that mortality rates were higher in studies that include patients with both PTB and EPTB than in studies that include patients with only PTB. This finding could be due to late diagnosis and treatment because early diagnosis is a challenge for EPTB cases. Extrapulmonary TB can also be associated with other confounding risk factors for death, such as immunosuppression and chronic illness.^37^

### Adverse Events

We found that adverse events were common among pregnant patients with MDR-TB, with more than half of the patients (54.7%) experiencing at least 1 type of drug-related adverse event. This finding is consistent with a previous systematic review that found that 57.3% of patients with MDR-TB experienced at least 1 kind of adverse event, with gastrointestinal disorders, ototoxicity, and psychiatric disorders being the most common.^38^ High rates of drug-associated adverse events in the treatment of patients with MDR-TB were also reported in a recent meta-analysis using individual patient data.^39^ The high frequency of adverse events suggests that pregnant patients receiving medication for MDR-TB should be monitored closely and managed aggressively for adverse effects. Data were not available in our systematic review to identify the type of adverse events associated with specific TB drugs.

Although different treatment regimens were used by pregnant patients, the most common types of TB medicine included in the regimens were pyrazinamide, cycloserine, prothionamide, and fluoroquinolones. It was not possible to assess the independent associations of these drugs with treatment outcomes because various regimens were used for the treatment of MDR-TB. Although some second-line TB medications, such as amikacin, streptomycin, prothionamide, and ethionamide, are considered teratogenic and are usually contraindicated for pregnant patients, these drugs were used in some of the included studies.

### Pregnancy Outcomes

Preterm birth, pregnancy loss, low birth weight, and stillbirth were the most common types of adverse pregnancy outcomes found in our systematic review. These findings are consistent with a previous systematic review among patients with drug-susceptible TB that reported that TB during pregnancy is associated with adverse pregnancy outcomes, such as low birth weight and stillbirth.^40^ The prevalence of preterm birth among patients with MDR-TB in our study (9.5%) was similar to the worldwide prevalence of preterm birth (11%), the prevalence of preterm birth among patients with drug-susceptible TB (8.0%), and the prevalence of preterm birth in a previous systematic review of MDR-TB among pregnant patients (12.9%).^4^ The prevalence of stillbirth in our study (1.9%) was also similar to that reported for patients with non-TB diseases, such as COVID-19 (1.4%),^41^ and among the general population (1.8%).^42,43^ However, the prevalence of low birth weight (3.9%) in our study was lower than the prevalence of low birth weight among patients with drug-susceptible TB (8.5%),^44^ in the general population (14.6%),^45,46^ and in a previous systematic review (23.6%). The reason for the difference in the prevalence of low birth weight between our systematic review and the previous systematic review could be the difference in the number of studies included in the systematic review. In the previous systematic review, only 2 studies were included, whereas in the present systematic review, 6 studies were included. The main reasons for pregnancy loss were miscarriage and therapeutic termination of pregnancy.

### Limitations

This study has some limitations. Because only 10 studies with a total of less than 300 participants met our inclusion criteria, the metaregression model was underpowered to detect associations between study characteristics and successful treatment outcomes. There were no studies from the Southeast Asia region, India, Russia, or other countries with a high TB burden. In addition, the crude classification of study characteristics, such as stratifying analyses at the median value for the proportion of patients receiving a certain drug, may have resulted in misclassification of exposure for many patients treated with individualized regimens and may have reduced our chances of finding any associations with outcomes, even if present. Treatment success rates did not differ by TB medicines (except for linezolid), but the small sample size and the lack of individual patient data prevent us from drawing firm conclusions. Moreover, owing to the limited number of studies, we were not able to stratify adverse drug events based on medication types. Selection bias could also be a major limitation of our study because almost all of the studies included in our systematic review were conducted using retrospectively collected data. This review highlights the need for more standardized reporting, as well as evidence from well-designed randomized clinical trials, or a meta-analysis of pooled individual patient data from multiple observational studies among pregnant patients with MDR-TB.

## Conclusions

The findings of this meta-analysis and systematic review suggest that high treatment success rates and favorable pregnancy outcomes can be achieved when pregnant patients with MDR-TB are treated with effective regimens. Further research is needed to design shorter, more effective, and safer treatment regimens for pregnant patients with MDR-TB.

## Notes

Supplement. **eTable 1.** Search Strategy **eTable 2.** Definition of Treatment Outcomes **eTable 3.** Quality Assessment Tool for Included Studies **eTable 4.** The World Health Organization (WHO) Grouping of Medicines Recommended for Use in Longer Multidrug-Resistant Tuberculosis (MDR-TB) Regimens **eTable 5.** Studies Excluded From the Systematic Review and Reason for Exclusion **eTable 6.** Characteristics of Included Studies **eTable 7.** Metaregression for Treatment Outcomes of Pregnant Patients With Multidrug-Resistant Tuberculosis **eTable 8.** Subgroup Analysis and Metaregression of Treatment Success Based on the Type of Tuberculosis Medicines **eTable 9.** Type of Drug-Related Adverse Events Reported Among Pregnant Patients With Multidrug-Resistant Tuberculosis **eTable 10.** Pregnancy Outcomes Among Patients With Multidrug-Resistant Tuberculosis **eTable 11.** Quality Assessment Score of the Studies Included in the Systematic Review and Meta-analysis **eFigure 1.** Pooled Percentage of Treatment Success Among Pregnant Patients With Multidrug-Resistant Tuberculosis **eFigure 2.** Pooled Percentage of Death Among Pregnant Patients With Multidrug-Resistant Tuberculosis **eFigure 3.** Pooled Percentage of Treatment Failure Among Pregnant Patients With Multidrug-Resistant Tuberculosis **eFigure 4.** Pooled Percentage of Lost to Follow-up Among Pregnant Patients With Multidrug-Resistant Tuberculosis **eFigure 5.** Funnel Plot for Successful Treatment Outcomes Using Random-Effect Meta-analysis
eReferences.
