# Supplementary material for: Treatment Outcomes Among Pregnant Patients With Multidrug-Resistant Tuberculosis: A Systematic Review and Meta-analysis
Source: JAMA Netw Open. 2022 Jun 10;5(6):e2216527. doi: 10.1001/jamanetworkopen.2022.16527 (PMC9187956; doi:10.1001/jamanetworkopen.2022.16527)
Supplement: Supplement. — eTable 1. Search Strategy eTable 2. Definition of Treatment Outcomes eTable 3. Quality Assessment Tool for Included Studies eTable 4. The World Health Organization (WHO) Grouping of Medicines Recommended for Use in Longer Multidrug-Resistant Tuberculosis (MDR-TB) Regimens eTable 5. Studies Excluded From the Systematic Review and Reason for Exclusion eTable 6. Characteristics of Included Studies eTable 7. Metaregression for Treatment Outcomes of Pregnant Patients With Multidrug-Resistant Tuberculosis eTable 8. Subgroup Analysis and Metaregression of Treatment Success Based on the Type of Tuberculosis Medicines eTable 9. Type of Drug-Related Adverse Events Reported Among Pregnant Patients With Multidrug-Resistant Tuberculosis eTable 10. Pregnancy Outcomes Among Patients With Multidrug-Resistant Tuberculosis eTable 11. Quality Assessment Score of the Studies Included in the Systematic Review and Meta-analysis eFigure 1. Pooled Percentage of Treatment Success Among Pregnant Patients With Multidrug-Resistant Tuberculosis eFigure 2. Pooled Percentage of Death Among Pregnant Patients With Multidrug-Resistant Tuberculosis eFigure 3. Pooled Percentage of Treatment Failure Among Pregnant Patients With Multidrug-Resistant Tuberculosis eFigure 4. Pooled Percentage of Lost to Follow-up Among Pregnant Patients With Multidrug-Resistant Tuberculosis eFigure 5. Funnel Plot for Successful Treatment Outcomes Using Random-Effect Meta-analysis eReferences. [file jamanetwopen-e2216527-s001.pdf]

Supplementary Online Content

Alene KA, Murray MB, van de Water BJ, et al. Treatment outcomes among pregnant patients with multidrug-resistant tuberculosis: a systematic review and meta-analysis. *JAMA Netw Open.* 2022;5(6):e2216527. doi:10.1001/jamanetworkopen.2022.16527

eTable 1. Search Strategy
eTable 2. Definition of Treatment Outcomes
eTable 3. Quality Assessment Tool for Included Studies
eTable 4. The World Health Organization (WHO) Grouping of Medicines Recommended for Use in Longer Multidrug-Resistant Tuberculosis (MDR-TB) Regimens
eTable 5. Studies Excluded From the Systematic Review and Reason for Exclusion
eTable 6. Characteristics of Included Studies
eTable 7. Metaregression for Treatment Outcomes of Pregnant Patients with Multidrug-Resistant Tuberculosis
eTable 8. Subgroup Analysis and Metaregression of Treatment Success Based on the Type of Tuberculosis Medicines
eTable 9. Type of Drug-Related Adverse Events Reported Among Pregnant Patients With Multidrug-Resistant Tuberculosis
eTable 10. Pregnancy Outcomes Among Patients With Multidrug-Resistant Tuberculosis
eTable 11. Quality Assessment Score of the Studies Included in the Systematic Review and Meta-analysis
eFigure 1. Pooled Percentage of Treatment Success Among Pregnant Patients With Multidrug-Resistant Tuberculosis
eFigure 2. Pooled Percentage of Death Among Pregnant Patients With Multidrug-Resistant Tuberculosis
eFigure 3. Pooled Percentage of Treatment Failure Among Pregnant Patients With Multidrug-Resistant Tuberculosis
eFigure 4. Pooled Percentage of Lost to Follow-up Among Pregnant Patients With Multidrug-Resistant Tuberculosis
eFigure 5. Funnel Plot for Successful Treatment Outcomes Using Random-Effect Meta-analysis
eReferences.

This supplementary material has been provided by the authors to give readers additional information about their work.

eTable 1. Search Strategy

We searched PubMed, SCOPUS, Web of Sciences, and ProQuest for studies published in any language up to and including 31 Aug 2021 that reported treatment outcomes of multidrug-resistant tuberculosis (MDR-TB) among pregnant women. We searched using keywords related to MDR-TB and pregnancy. Full search strategies for each database are provided below in a table. The search was conducted in English but without language restriction.

PubMed			
Number	Themes	Searching query	Items
1	MDR/XDR TB	"Tuberculosis, multidrug resistant"[MeSH Terms] OR "multidrug resistant tuberculosis"[Title/Abstract] OR "drug resistant tuberculosis"[Title/Abstract] OR "multiple drug resistant tuberculosis"[Title/Abstract] OR "mdr tuberculosis"[Title/Abstract] OR "mdr tb"[Title/Abstract] OR "MDRTB"[Title/Abstract] OR "extensively drug resistant tuberculosis"[Title/Abstract] OR "XDR TB" [Title/Abstract] OR "XDRTB" [Title/Abstract] OR (("drug resistance"[Title/Abstract] OR "multidrug resistance"[Title/Abstract] OR "multiple drug resistance"[Title/Abstract] OR "multiresistant"[Title/Abstract] OR "multi resistant"[Title/Abstract]) AND ("tuberculosis"[Title/Abstract] OR "TB"[Title/Abstract]))	16,589
2	Pregnancy	"pregnancy"[MeSH Terms] OR "pregnan*"[Title/Abstract] OR "obstetric*"[Title/Abstract] OR "wom"[Title/Abstract] OR "gestation"[Title/Abstract]	1,116,873
3	Search	#1 AND #2	144
4	Limit (human)		**128**
SCOPUS			
1	MDR/XDR TB	(Multidrug AND resistant AND tuberculosis) OR (tuberculosis AND multidrug-resistant) OR (multidrug AND resistant AND TB) OR (extensive AND drug AND resistant AND tuberculosis) OR mdrtb OR mdr-tb OR xdrtb OR xdr-tb	16,574
2	Pregnancy	pregnancy OR pregnant OR obstetric women OR gestation	439,538
3	Search	#1 AND #2	**95**
Web of Sciences			
1	MDR/XDR TB	Multidrug Resistant Tuberculosis (Topic) or multiple drug resistant tuberculosis (Topic) or extensively drug resistant tuberculosis (Topic) or Extensively Drug-Resistant Tuberculosis (All Fields) or Multidrug-resistant Tuberculosis (Topic) or MDR-TB (All Fields) or XDR-TB (Topic) or mdr tuberculosis (Topic) or mdr tuberculosis (Topic)	13,194
2	Pregnancy	((((TS=(pregnancy)) OR TS=(pregnant)) OR TS=(obstetric)) OR TS=(gestation)) OR TS=(women)	1,965,713
3	Search	(#2) AND #3	**232**
ProQuest			
1	MDR/XDR TB	(ti("Multidrug Resistant Tuberculosis") OR ti("multiple drug resistant tuberculosis") OR ti("extensively drug resistant tuberculosis") OR ti("Extensively Drug-Resistant Tuberculosis") OR ti("Multidrug-resistant Tuberculosis") OR ti(mdr-tb) OR ti(xdr-tb) OR ti("mdr tuberculosis") OR ti("xdr tuberculosis"))	6,198
2	Pregnancy	ti(pregnancy) OR ti(pregnant) OR ti(obstetric) OR ti(gestation) OR ti(women)	6,690,470
3	Search	#1 AND #2	**32**

© 2022 Alene KA et al. *JAMA Network Open.*

eTable 2. Definition of Treatment Outcomes

The definitions of multidrug-resistant tuberculosis (MDR-TB) treatment outcomes such as cure, treatment completion, death, loss to follow up and treatment failure were based on the World Health Organization (WHO) guidelines[1]. Treatment success was defined as the sum of cure and treatment completion. Poore treatment outcomes were defined as the sum of death, loss to follow-up and treatment failure.

eTable 2. Treatment outcomes of MDR-TB were defined the WHO guideline

Treatment outcomes	Definitions
Cure	Completed MDRTB therapy with five or more negative cultures in the last 12 months of treatment; alternatively, a participant could have one positive culture followed by at least three negative cultures separated by 30 days with no clinical deterioration.
Treatment completion	Completed MDRTB therapy without meeting the definition of cure
Death	Mortality during MDR-TB treatment from any cause
Loss to follow-up	Interruption of therapy for ≥2 consecutive months for any reason.
Treatment failure	Two of five cultures were positive within the last 12 months of therapy or any culture positivity within the last three cultures; alternatively, failure was defined as treatment discontinuation due to lack of appropriate response or significant adverse events.

© 2022 Alene KA et al. *JAMA Network Open.*

eTable 3. Quality Assessment Tool for Included Studies

Quality assessment of individual studies was done using the Newcastle-Ottawa scale. The three aspects of the included study assessed using this scale were a selection of study groups (maximum 5 points), comparability or quality of adjustment for confounding factors (maximum 1 point), and ascertainment of the outcome of interest (maximum 3 points). The maximum number of points was 9, representing the highest methodological quality.

**Selection: (maximum 5 points)**	
**Study Population**	
1	The study population is clearly defined
0	The study population is not clearly defined
**Representativeness of exposed cohort**	
2	The study sample is representative of the exposed population (all pregnant women or random sampling of pregnant women)
1	The study sample comprises a select group of the study population (non-random sampling)
0	No description of the sampling strategy.
**Ascertainment of exposure**	
1	The study clearly defines the exposure (i.e., clear definition for MDR-TB OR second-line TB medications)
0	The study does not clearly define the exposure
**Sample size**	
1	Justified and satisfactory (sample size and power calculation included)
0	Not justified
**Non-respondents**	
1	Comparability between respondents and non-respondents' characteristics is established, and the response rate is satisfactory.
0	The response rate is unsatisfactory, or the comparability between respondents and non-respondents is unsatisfactory; OR no description of the response rate or the characteristics of the responders and the non-responders.
**Comparability: (maximum 1 point)**	
**Impact of Bias (selection bias, measurement bias, participant reporting, confounders)**	
1	Where appropriate the study acknowledges and mitigates for potential bias (i.e., when comparisons are made between different study populations results are normalized for confounders)
0	Where appropriate the study does not acknowledge or mitigate for potential bias.
**Outcome: (maximum 3 points)**	
**Assessment of the outcome (i.e., treatment outcomes)**	
1	Objective assessment tools or definitive diagnostic methods.
0	No definitive assessment or diagnostic tools or self-report
**Statistical analysis**	

© 2022 Alene KA et al. *JAMA Network Open.*

1	The statistical test used is clearly described and appropriate. Where comparisons are made between population groups, the measurement of the association is presented, including confidence intervals and the probability level (p-value)
0	The statistical test is inappropriate/not described/incomplete
**Adequacy of follow up of the cohort**	
1	Follow-up was judged adequate if the treatment outcomes were recorded for all patients
0	Follow-up was judged inadequate if the treatment outcomes were not recorded for patients due to transfer out or still on treatment.

© 2022 Alene KA et al. *JAMA Network Open.*

eTable 4. The World Health Organization (WHO) Grouping of Medicines Recommended for Use in Longer Multidrug-Resistant Tuberculosis (MDR-TB) Regimens[1]

Groups	Medicines
Group A	Levofloxacin/Moxifloxacin
	Bedaquiline
	Linezolid
Group B	Clofazimine
	Cycloserine/Terizidone
Group C	Ethambutol
	Delamanid
	Pyrazinamide
	Imipenem–cilastatin/ Meropenem
	Amikacin/Streptomycin
	Ethionamide/ Prothionamide
	P-aminosalicylic acid

© 2022 Alene KA et al. *JAMA Network Open.*

eTable 5. Studies Excluded From the Systematic Review and Reason for Exclusion

Our electronic database searches identified 487 records. After removal of duplicates, 366 unique records were screened by title and abstract, which result in 23 potential articles for full-text review. Based on the full-text review, 13 studies were excluded. The excluded studies and the reason for exclusion are presented in the table below.

SN	Study	Reason of exclusion
1.	Asgeirsson H, 2009	Review article
2.	Majori M., 2014	No data on treatment outcome
3.	Nesterenko AV, 2017	No data on MDR-TB treatment outcome for pregnant women
4.	Nesterenko AV, 2018	No treatment outcome data for MDR-TB pregnant women
5.	Takashima T, 2006	Case series with less than five pregnant women (i.e., only 3 cases)
6.	Helbling H, 2014	No data on treatment outcomes for pregnant women
7.	Loveday M, 2019	No data on treatment outcome
8.	Mesic, A. 2020	No data on treatment outcomes for pregnant women
9.	Nitta AT, 1999	Case series with less than five pregnant women (i.e., only 4 cases)
10.	Dudnyk A, 2016	Poster abstracts with insufficient data
11.	Baluku JB, 2021	No data for treatment outcome for pregnant women
12.	Desai U, 2018	Case series with less than five pregnant women (i.e., only 4 cases)
13.	Khan M, 2007	Case series with less than five pregnant women (i.e., only 3 cases)

© 2022 Alene KA et al. *JAMA Network Open.*

eTable 6. Characteristics of Included Studies

The affected body site was not reported in half of the studies. Three studies included pulmonary DR-TB only and two studies included both pulmonary and extra-pulmonary DR-TB[2,3]. While all studies reported their study population as having MDR-TB, five studies included patients with extensively drug-resistant TB (XDR-TB)[2,4-6] and four studies included rifampicin-resistant TB (RR-TB, in the absence of INH resistance)[2,5,7,8]. Seven studies reported the HIV status of pregnant women[2-5,9-11]. While one study included HIV co-infected patients only[10], the proportion of HIV infected women in the other studies varied from 28.5% to 81.0%[4]. Data on ART use was reported in four of these studies[2,4,5,9], with the percentage of ART use varying from 60% to 100%[9]. Eight studies reported the timing of pregnancy during MDR-TB treatment[2-6,8,9,11]. Four of these studies reported that more than half of the women were pregnant before the start of treatment[2-4,8].

Study	Country	Study design	Study period	Age*	Sample size	TB treatment history (%)	HIV (%)
Mokhele (2021)[2]	South Africa	Retrospective cohort	2010-2016	30	35	37.1	68.5
Loveday (2021)[4]	South Africa	Retrospective cohort	2013-2017	28	108	46	81
Baluku (2021)[9]	Uganda	Retrospective cohort	2013-2019	27.5	18	66.7	44.4
van der Walt (2020)[5]	South Africa	Retrospective cohort	2010 -2018	29	26	57.7	76.9
van de Water (2020)[7]	Peru	Prospective cohort	2009-2012	26	8	37.5	NA
Azeez (2018)[10]	South Africa	Retrospective cohort	2010-2016	37	36	NA	100
Tabarsi (2011)[8]	Iran	Retrospective cohort	2003-2009	22	5	NA	NA
Oliveira (2011)[11]	Brazil	Retrospective cohort	1995-2007	25	7	NA	28.5
Palacios (2009)[3]	Peru	Retrospective cohort	1996-2005	24.4	38	89.5	7.9
Shin (2003)[6]	Peru	Retrospective cohort	1996-2003	21	7	NA	NA

*Mean or median age in a year; NA: not available

© 2022 Alene KA et al. *JAMA Network Open.*

eTable 7. Metaregression for Treatment Outcomes of Pregnant Patients with Multidrug-Resistant Tuberculosis

Categories	Treatment success		Death		Failure		Lost to follow up	
	OR (95% CI)	P value	OR (95% CI)	P value	OR (95% CI)	P value	OR (95% CI)	P value
**Country**								
South Africa	1.00		1.00		1.00		1.00	
Peru	1.001 (0.82, 1.21)	0.98	1.04 (0.95, 1.15)	0.27	1.03 (0.97, 1.10)	0.16	**0.88 (0.82, 0.94)**	**0.005**
Others*	1.04 (0.71, 1.52)	0.77	1.07 (0.79, 1.45)	0.58	**0.95 (0.96, 0.99)**	**0.009**	**0.86 (0.79, 0.93)**	**0.005**
**Study period**								
2010 – 2019	1.00		1.00		1.00		1.00	
1996 – 2009	0.91 (0.73, 1.15)	0.40	1.12 (0.96, 1.31)	0.11	1.03 (0.98, 1.09)	0.11	**0.89 (0.82, 0.96)**	**0.01**
**Median age^**								
21 – 25 years	1.00		1.00		1.00		1.00	
26 – 30 years	1.08 (0.86, 1.36)	0.40	0.88 (0.76, 1.03)	0.11	0.96 (0.91, 1.01)	0.11	**1.11 (1.03, 1.21)**	**0.01**
**Body sites**								
PTB only	1.00	1.00	1.00		1.00		1.00	
Both PTB and EPTB	0.94 (0.84, 1.04)	0.22	**1.07 (1.01, 1.13)**	**0.02**	1.003 (0.94, 1.06)	0.88	0.97 (0.83, 1.13)	0.69
Unknown	1.08 (0.86, 1.36)	0.43	1.10 (0.85, 1.42)	0.39	1.002 (0.91, 1.10)	0.95	**0.86 (0.79, 0.94)**	**0.006**
**Previous TB treatment**								
≤ 50%	1.00		1.00		1.00		1.00	
> 50 %	1.02 (0.87, 1.20)	0.71	1.01 (0.91, 1.11)	0.80	1.01 (0.95, 1.06)	0.35	0 .94 (0.83, 1.06)	0.26
Unknown	1.07 (0.79, 1.46)	0.58	1.18 (0.82, 1.71)	0.29	1.04 (0.88, 1.22)	0.54	**0.84 (0.72, 0.97)**	**0.02**
**HIV prevalence**								
≤ 50%	1.00		1.00		1.00		1.00	
> 50 %	1.03 (0.79, 1.33)	0.77	0.91 (0.77, 1.09)	0.26	0.99 (0.93, 1.04)	0.70	**1.11 (1.06, 1.17)**	**0.001**
Unknown	1.17 (0.84, 1.63)	0.27	0.90 (0.71, 1.13)	0.30	1.02 (0.87, 1.19)	0.73	0.92 (0.82, 1.03)	0.13

© 2022 Alene KA et al. *JAMA Network Open.*

eTable 8. Subgroup Analysis and Metaregression of Treatment Success Based on the Type of Tuberculosis Medicines

The median value was used to categorise the variables.

Group*	Medicines^	Number of studies	Pooled treatment success % (95% CI)	OR (95% CI)	P value
Group A	Patients who received levofloxacin				
	⩽51.5%	2	73.2 (58.7, 85.7)	1.00	
	>51.5%	2	69.0 (59.8, 77.7)	0.97 (0.59, 1.59)	0.86
	Patients who received linezolid				
	⩽20.1%	2	65.6 (57.3, 73.5)	1.00	
	>20.1%	2	85.0 (67.4, 97.3)	**1.22 (1.05, 1.42)**	**0.027**
Group B	Patients who received clofazimine				
	⩽20.1%	3	72.5 (54.1, 87.9)	1.00	
	>20.1%	3	70.6 (60.2, 80.1)	1.005 (0.80, 1.26)	0.947
	Patients who received cycloserine/terizidone				
	⩽79.2%	3	67.0 (50.5, 81.8)	1.00	
	>79.2%	5	71.2 (61.8, 79.8)	1.03 (0.84, 1.27)	0.43
Group C	Patients who received ethambutol				
	⩽29.0%	3	65.6 (58.7, 72.2)	1.00	
	>29.0%	3	82.1 (64.9, 95.1)	1.16 (0.994, 1.35)	0.056
	Patients who received pyrazinamide				
	<78.1%	4	64.3 (53.7, 74.3)	1.00	
	⩾78.1%	4	80.4 (64.3, 93.1)	1.14 (0 .95, 1.36)	0.113
	Patients who received ethionamide/protionamide				
	⩽65.8%	3	82.1 (64.9, 95.1)	1.00	0.318
	>65.8%	3	72.5 (54.1, 87.9)	0 .90 (0 .71, 1.14)	
	Patients who received PAS				
	⩽49.6%	3	66.7 (53.8, 78.4)	1.00	
	>49.6%	3	72.0 (58.4, 84.1)	1.03 (0 .81, 1.32)	0.707
	Patients who received amikacin				
	⩽43.2%	2	65.9 (49.2, 81.1)	1.00	

© 2022 Alene KA et al. *JAMA Network Open.*

Group*	Medicines^	Number of studies	Pooled treatment success % (95% CI)	OR (95% CI)	P value
	>43.2%	2	70.2 (60.5, 79.1)	1.07 (0 .72, 1.61)	0.498
Other medicines	Patients who received capreomycin				
	⩽28.3%	3	65.6 (57.9, 72.9)	1.00	0.394
	>28.3%	3	72.0 (58.4, 84.1)	1.06 (0 .88, 1.28)	
	Patients who received isoniazid				
	⩽22.2%	3	66.4 (59.0, 73.5)	1.00	0.314
	>22.2%	2	80.0 (59.3, 95.5)	1.11 (0 .84, 1.46)	
	Patients who received ofloxacin				
	⩽20.0%	2	73.2 (58.7, 85.7)	1.00	
	>20.0%	2	86.0 (54.1100.0)	1.06 (0 .416, 2.73)	0.792
	Patients who received kanamycin				
	⩽29.0%	2	65.3 (46.2, 82.5)	1.00	0.846
	>29.0%	2	63.5 (51.0, 75.2)	0.99 (0 .86, 1.13)	

^Studies that had no clear information of how many participants used the drug were excluded from this sub-group analysis. The median was used to categorise the variable. PAS: Para-aminosalicylic acid. * Based on WHO grouping.

© 2022 Alene KA et al. *JAMA Network Open.*

eTable 9. Type of Drug-Related Adverse Events Reported Among Pregnant Patients With Multidrug-Resistant Tuberculosis

Type of adverse events	Number of included studies	Total number of patients	Number with adverse event	Proportions of adverse events (95% CI)
No adverse events	4 studies[4-6,9]	86	39	45.3 (34.5, 56.4)
Women with at least one adverse event	4 studies[4-6,9]	86	47	54.7 (43.5, 65.4)
Gastrointestinal disorders	3 studies[4-6]	68	8	11.8 (5.2, 21.8)
Hearing loss	3 studies[4-6]	76	9	11.8 (5.5, 21.3)
Anaemia	3 studies[4-6]	79	7	8.9 (3.6, 17.4)
Dizziness	2 studies[4,5]	61	4	6.6 (1.8, 15.9)
Psychiatric disorder	2 studies[5,6]	44	4	9.1 (2.5, 21.6)
Hypokalaemia	2 studies[4,6]	42	5	11.9 (3.9, 25.6)
Skin rash	2 studies[4,5]	61	3	4.9 (1.0, 13.7)
Liver function impairment	2 studies[4,9]	46	14	30.4 (17.7, 45.7)
Renal function impairment	2 studies[4,9]	47	7	14.9 (6.2, 28.3)

Adverse events reported only by one study were not included in this pooled proportion.

© 2022 Alene KA et al. *JAMA Network Open.*

eTable 10. Pregnancy Outcomes Among Patients With Multidrug-Resistant Tuberculosis

Study	Sample size	Unknown pregnancy outcomes	Known pregnancy outcomes	Favourable pregnancy outcomes	Adverse pregnancy outcomes						
					Preterm birth	Miscarriage or abortion	Neonatal death	Termination of pregnancy	Stillbirth	Low birthweight	Pregnancy loss^
Mokhele (2021)	35	15	20	4	11	1	1	3	0	0	4
Loveday (2021) *	108	0	109	57	28	3	NA	1	6	33	4
van der Walt (2020)	26	1	25	22	0	2	0	0	1	0	2
Tabarsi (2011)	5	0	5	5	0	0	0	0	0	0	0
Palacios (2009)	38	0	38	28	1	5	0	0	1	3	5
Shin (2003)	7	0	7	7	0	0	0	0	0	0	0

*108 pregnant women with 109 foetuses, including a set of twins. ^Total pregnancy loss is the sum of miscarriage and termination of pregnancy. Total adverse outcomes: 29.1 (6.3, 58.3). NA: not available

© 2022 Alene KA et al. *JAMA Network Open.*

eTable 11. Quality Assessment Score of the Studies Included in the Systematic Review and Meta-analysis

References	Selection					Comparability	Outcome			Total Score
	Study Population	Representativeness	Ascertainment	Sample size	Non-response	Comparability of the cohorts^	Assessment of the outcome	Statistical analysis	Adequacy of follow up of the cohort	
Mokhele (2021)[2]	1	1	1	1	0	1	1	1	0	7
Loveday (2021)[4]	1	1	1	1	0	1	1	1	1	8
Baluku (2021)[9]	1	1	1	0	0	1	1	0	1	6
van der Walt (2020)[5]	1	1	1	0	0	1	1	1	1	7
van de Water (2020)[7]	1	1	1	0	1	1	1	1	1	8
Azeez (2018)[10]	1	1	1	1	0	1	1	1	1	8
Tabarsi (2011)[8]	1	0	1	0	0	0	1	0	1	4
Oliveira (2011)[11]	1	0	1	0	0	0	1	0	0	3
Palacios (2009)[3]	1	1	1	1	0	1	1	1	0	7
Shin (2003)[6]	1	0	1	0	0	1	1	0	0	4

^Comparability of the cohorts based on design or analysis

© 2022 Alene KA et al. *JAMA Network Open.*

eFigure 1. Pooled Percentage of Treatment Success Among Pregnant Patients With Multidrug-Resistant Tuberculosis

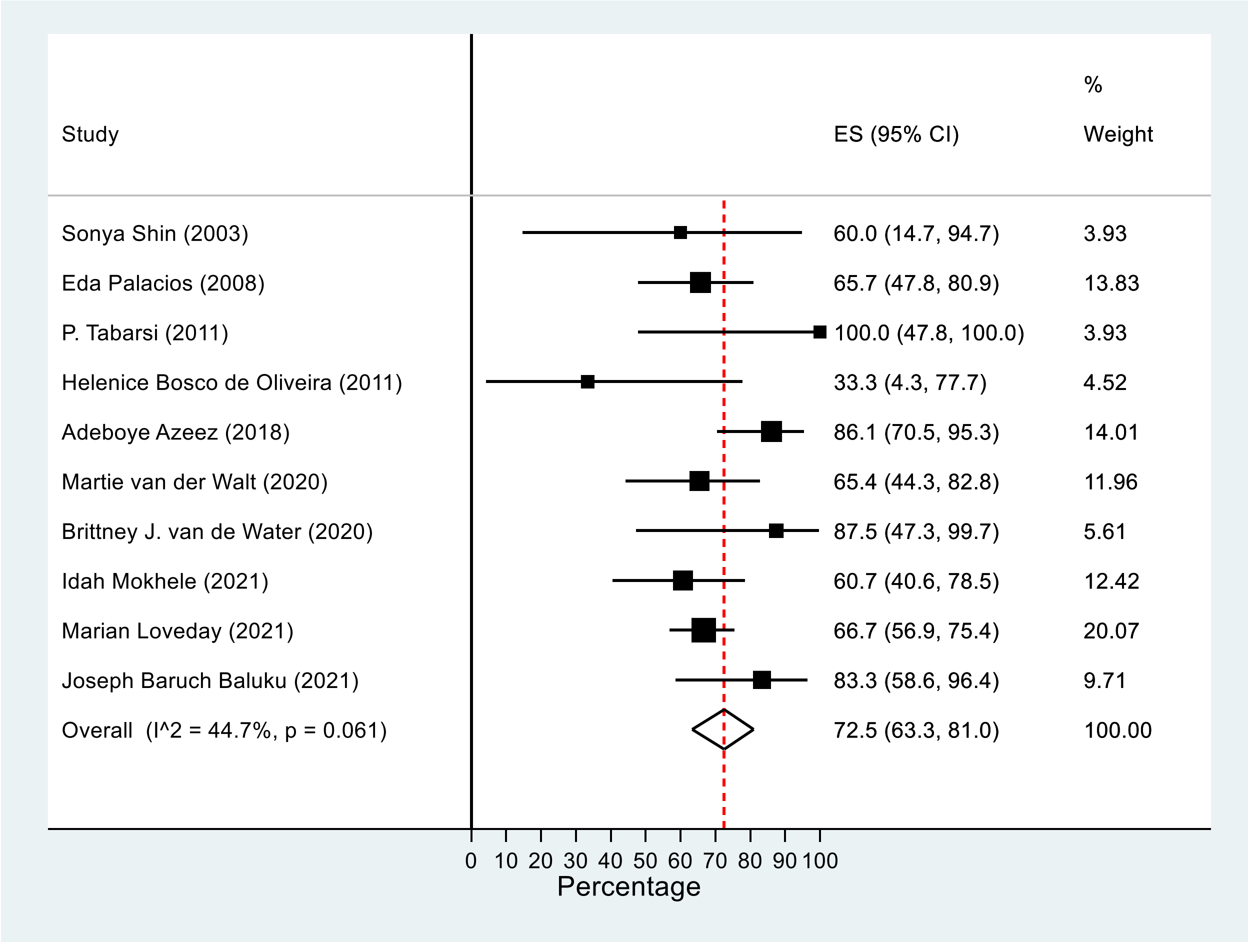

© 2022 Alene KA et al. *JAMA Network Open.*

eFigure 2. Pooled Percentage of Death Among Pregnant Patients With Multidrug-Resistant Tuberculosis

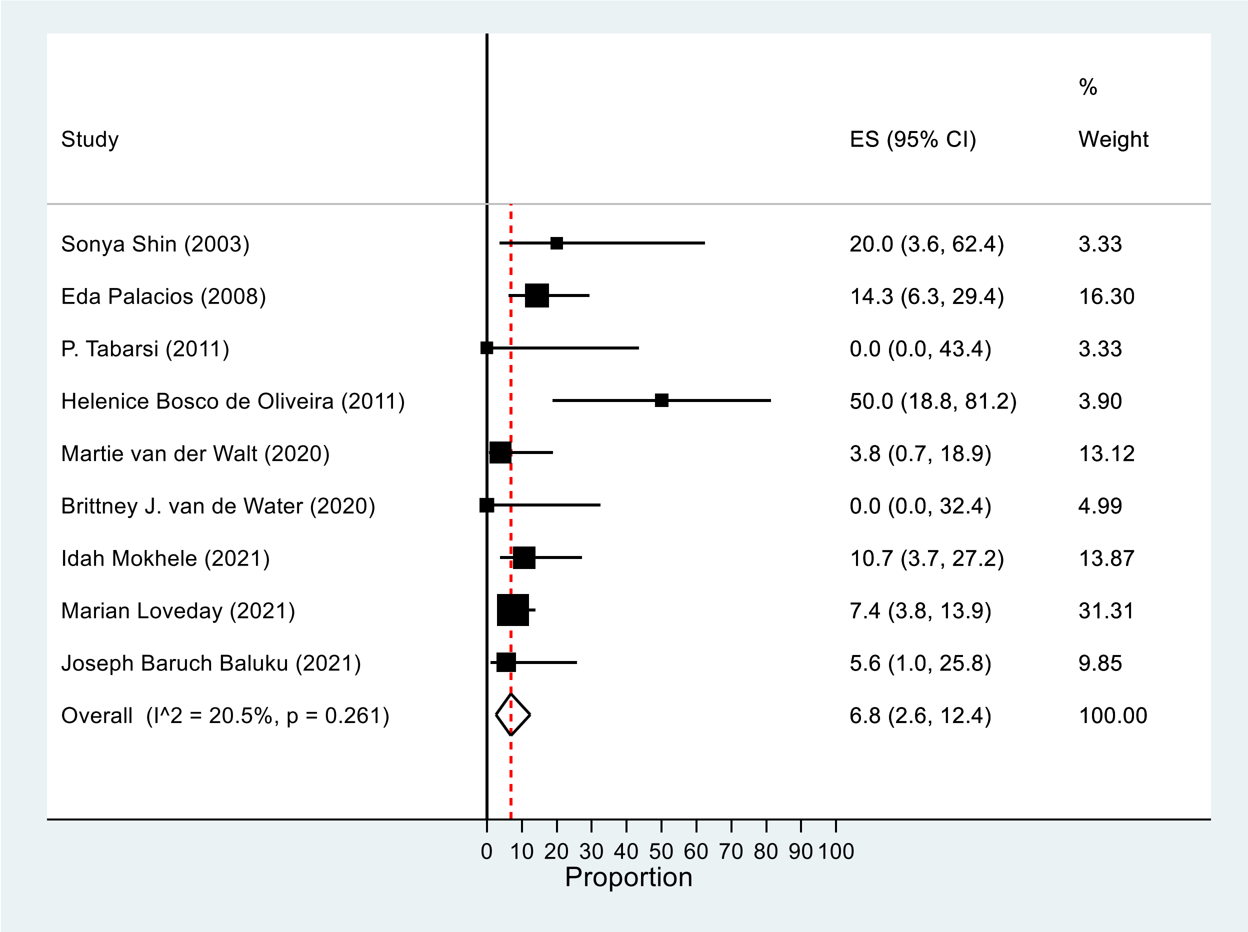

© 2022 Alene KA et al. *JAMA Network Open.*

eFigure 3. Pooled Percentage of Treatment Failure Among Pregnant Patients With Multidrug-Resistant Tuberculosis

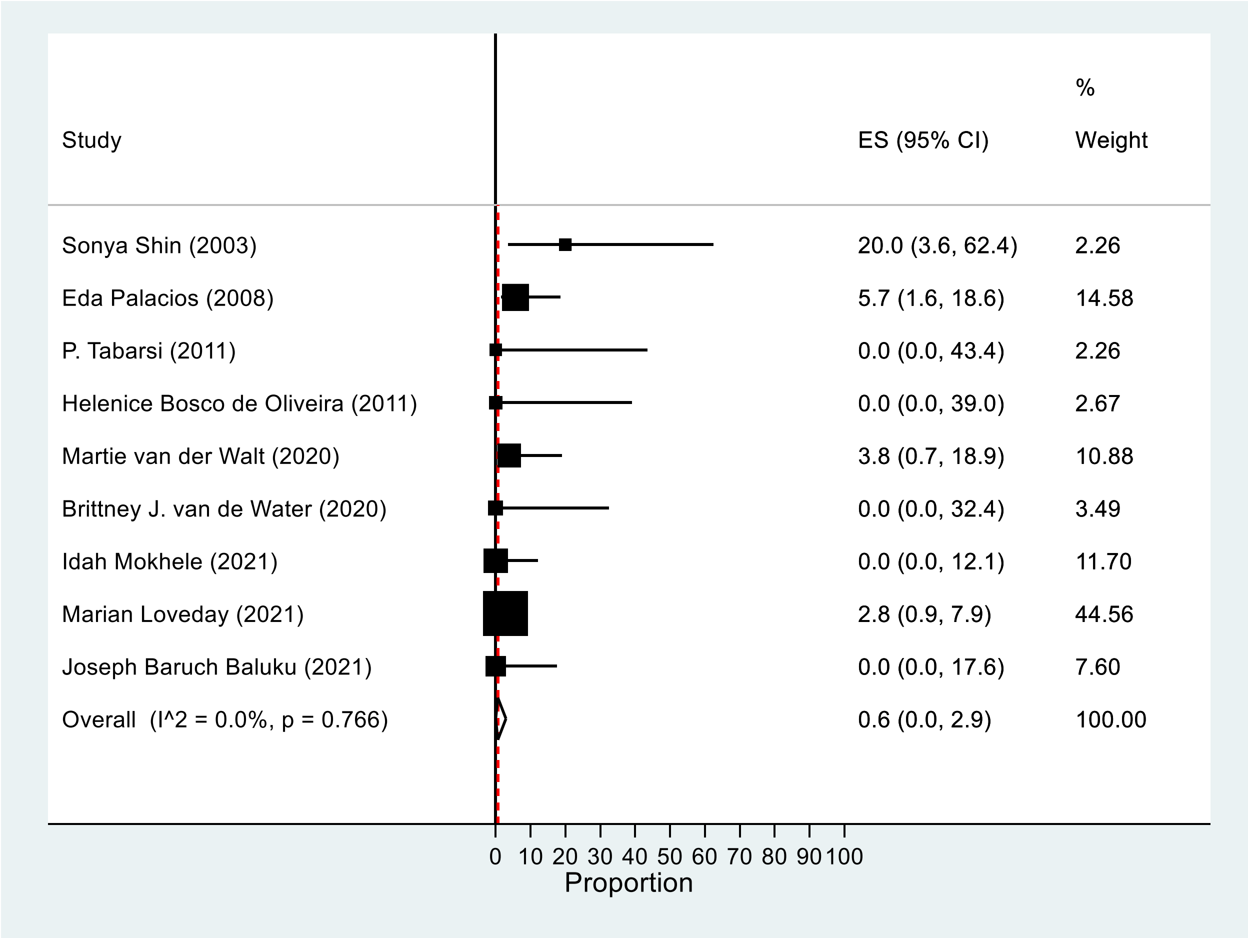

© 2022 Alene KA et al. *JAMA Network Open.*

eFigure 4. Pooled Percentage of Lost to Follow-up Among Pregnant Patients With Multidrug-Resistant Tuberculosis

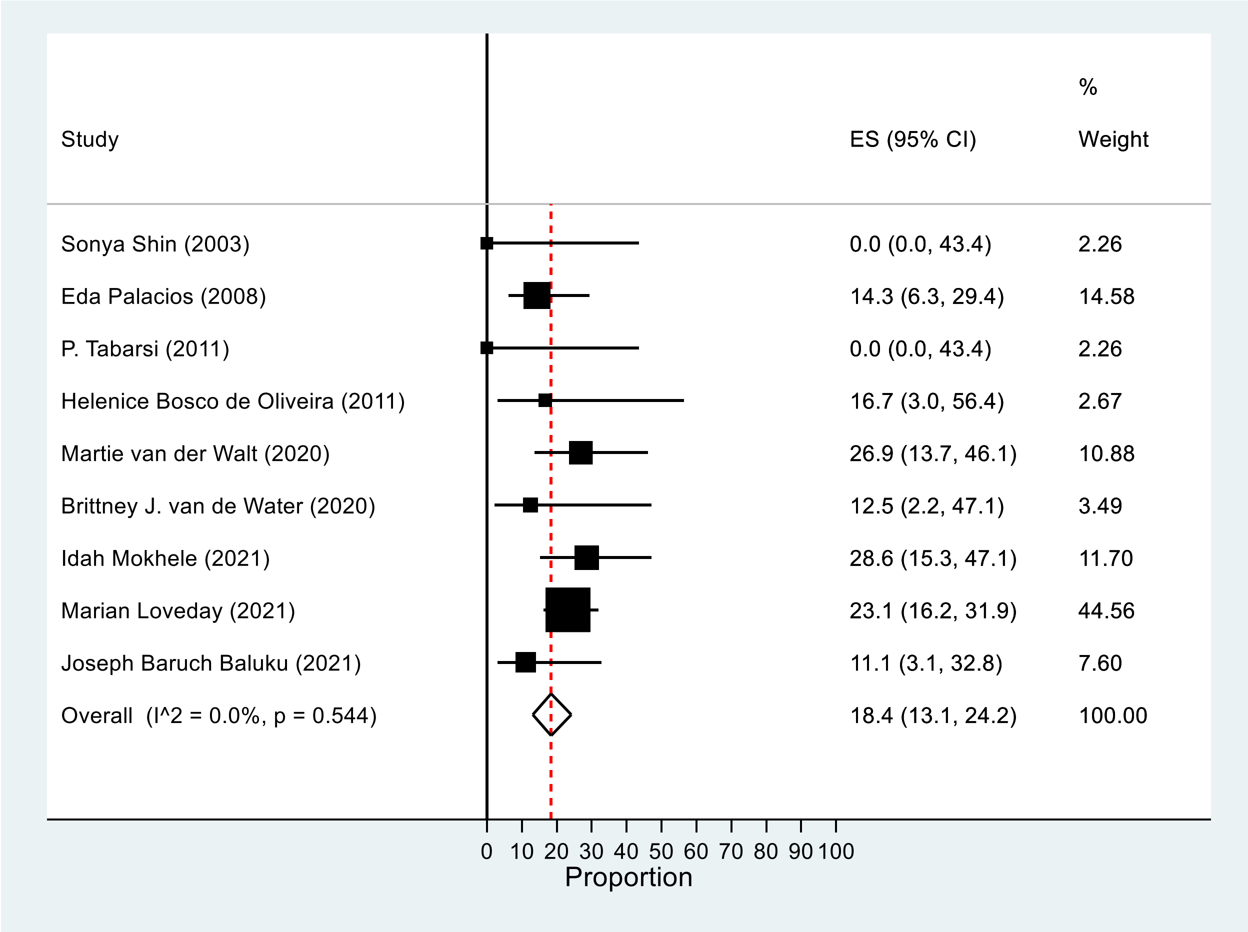

© 2022 Alene KA et al. *JAMA Network Open.*

eFigure 5. Funnel Plot for Successful Treatment Outcomes Using Random-Effect Meta-analysis

Egger's test for small-study effects gave a bias coefficient of 0.16 (95% CI: -0.89, 1.23) and a p-value of 0.72 indicating no significant publication bias.

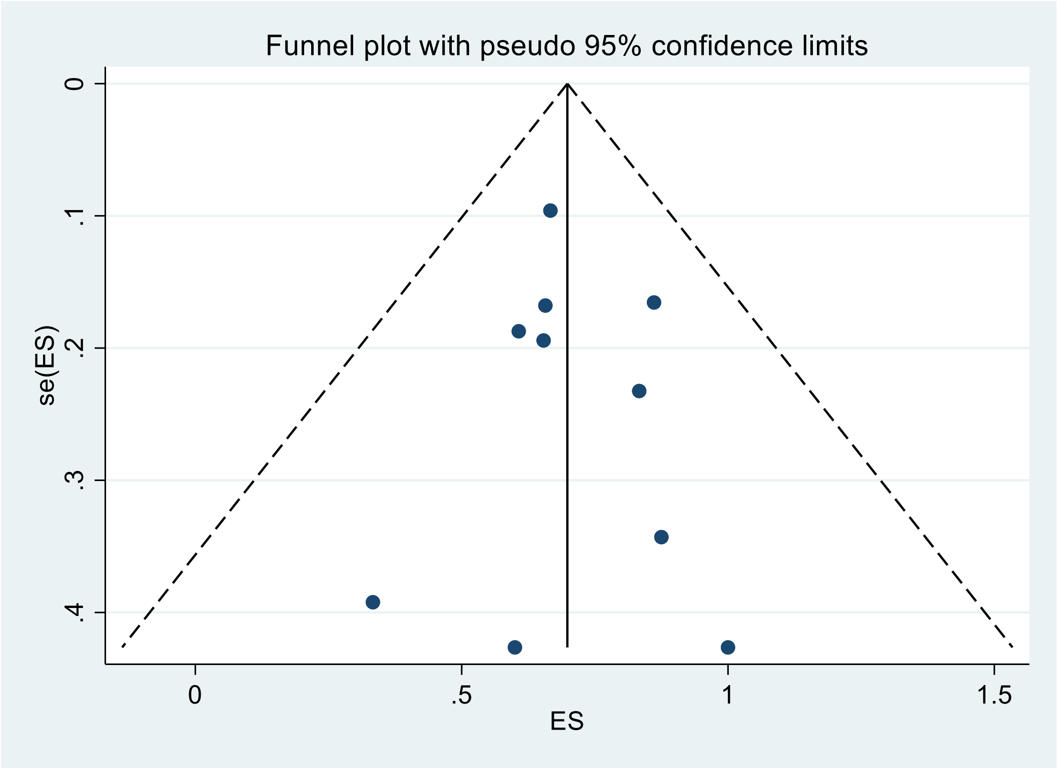

© 2022 Alene KA et al. *JAMA Network Open.*

eReferences.

1. WHO. *WHO consolidated guidelines on drug-resistant tuberculosis treatment.* Geneva: World Health Organization; 2019.
2. Mokhele I, Jinga N, Berhanu R, Dlamini T, Long L, Evans D. Treatment and pregnancy outcomes of pregnant women exposed to second-line anti-tuberculosis drugs in South Africa. *Bmc Pregnancy and Childbirth.* 2021;21(1).
3. Palacios E, Dallman R, Muñoz M, et al. Drug-resistant tuberculosis and pregnancy: treatment outcomes of 38 cases in Lima, Peru. *Clin Infect Dis.* 2008;48(10):1413-1419.
4. Loveday M, Hughes J, Sunkari B, et al. Maternal and Infant Outcomes Among Pregnant Women Treated for Multidrug/Rifampicin-Resistant Tuberculosis in South Africa. *Clin Infect Dis.* 2021;72(7):1158-1168.
5. Walt MV, Masuku S, Botha S, Nkwenika T, Keddy KH. Retrospective record review of pregnant women treated for rifampicin-resistant tuberculosis in South Africa. *PLoS One.* 2020;15(9):e0239018.
6. Shin S, Guerra D, Rich M, et al. Treatment of multidrug-resistant tuberculosis during pregnancy: a report of 7 cases. *Clin Infect Dis.* 2003;36(8):996-1003.
7. Van De Water BJ, Brooks MB, Huang CC, et al. Tuberculosis clinical presentation and treatment outcomes in pregnancy: A prospective cohort study. *BMC Infectious Diseases.* 2020;20(1).
8. Tabarsi P, Moradi A, Baghaei P, et al. Standardised second-line treatment of multidrug-resistant tuberculosis during pregnancy. *Int J Tuberc Lung Dis.* 2011;15(4):547-550.
9. Baluku JB, Bongomin F. Treatment outcomes of pregnant women with drug-resistant tuberculosis in Uganda: A retrospective review of 18 cases. *Int J Infect Dis.* 2021;105:230-233.
10. Azeez A, Ndege J, Mutambayi R. Associated factors with unsuccessful tuberculosis treatment outcomes among tuberculosis/HIV coinfected patients with drug-resistant tuberculosis. *Int J Mycobacteriol.* 2018;7(4):347-354.
11. Oliveira HBd, Mateus SHR. Caracterização da tuberculose multidroga resistente durante a gravidez, em Campinas, Estado de São Paulo, Brasil, no período de 1995 a 2007. *Revista da Sociedade Brasileira de Medicina Tropical.* 2011;44(5):627-630.

© 2022 Alene KA et al. *JAMA Network Open.*
